# A Novel Wheat C-bZIP Gene, *TabZIP14-B*, Participates in Salt and Freezing Tolerance in Transgenic Plants

**DOI:** 10.3389/fpls.2017.00710

**Published:** 2017-05-09

**Authors:** Lina Zhang, Lichao Zhang, Chuan Xia, Lifeng Gao, Chenyang Hao, Guangyao Zhao, Jizeng Jia, Xiuying Kong

**Affiliations:** ^1^School of Life Science, Northwest Normal UniversityLanzhou, China; ^2^Key Laboratory of Crop Gene Resources and Germplasm Enhancement, Ministry of Agriculture, The National Key Facility for Crop Gene Resources and Genetic Improvement, Institute of Crop Science, Chinese Academy of Agricultural SciencesBeijing, China

**Keywords:** wheat, abiotic stress, group C-bZIP TFs, TabZIP14-B, ABRE

## Abstract

The group C-bZIP transcription factors (TFs) are involved in diverse biological processes, such as the regulation of seed storage protein (SSP) production and the responses to pathogen challenge and abiotic stress. However, our knowledge of the abiotic functions of group C-bZIP genes in wheat remains limited. Here, we present the function of a novel *TabZIP14-B* gene in wheat. This gene belongs to the group C-bZIP TFs and contains six exons and five introns; three haplotypes were identified among accessions of tetraploid and hexaploid wheat. A subcellular localization analysis indicated that TabZIP14-B was targeted to the nucleus of tobacco epidermal cells. A transactivation assay demonstrated that TabZIP14-B showed transcriptional activation ability and was capable of binding the abscisic acid (ABA) responsive element (ABRE) in yeast. RT-qPCR revealed that *TabZIP14-B* was expressed in the roots, stems, leaves, and young spikes and was up-regulated by exogenous ABA, salt, low-temperature, and polyethylene glycol (PEG) stress treatments. Furthermore, *Arabidopsis* plants overexpressing *TabZIP14-B* exhibited enhanced tolerance to salt, freezing stresses and ABA sensitivity. Overexpression of *TabZIP14-B* resulted in increased expression of the *AtRD29A*, *AtCOR47*, *AtRD20*, *AtGSTF6*, and *AtRAB18* genes and changes in several physiological characteristics. These results suggest that *TabZIP14-B* could function as a positive regulator in mediating the abiotic stress response.

## Introduction

Wheat is widely cultivated around the world. Unfavorable conditions restrict wheat growth, development, and yields. Thus, the improvement of abiotic tolerance is a significant challenge in wheat breeding program, and novel gene discovery and utilization are critical for improving the tolerance of wheat to adverse conditions. Transcription factors (TFs) are vital regulators that mediate abiotic stress signal transduction. The basic leucine zipper (bZIP) TF is a large family characterized by a conserved bZIP domain with a basic region and a leucine zipper. Proteins of the bZIP family can bind to ACGT *cis*-elements, including abscisic acid (ABA) responsive element (ABRE) and G-box, C-box, and A-box elements (Izawa et al., 1993). The bZIP TF family is divided into 10 groups in *Arabidopsis* (Corrêa et al., 2008). Groups A, C, and S of bZIP TFs are involved in plant stress responses (Hu et al., 2016). Members of group A have been widely studied and are considered to be involved in ABA and stress signaling; these proteins include AtbZIP35/ABF1, AtbZIP36/ABF2/AREB1, AtbZIP37/ABF3, AtbZIP38/ABF4/AREB2, AtbZIP39, AtbZIP66/AREB3, and AtbZIP40 (Kim et al., 2004; Fujita et al., 2005; Furihata et al., 2006). The *TaAREB3* and *TabZIP60* genes belong to the AREB/ABF subgroup of group A. The overexpression of *TaAREB3* and *TabZIP60* in *Arabidopsis* significantly strengthens tolerance to several abiotic stresses and increases plant sensitivity to ABA (Zhang et al., 2015; Wang et al., 2016). All group C members of *Arabidopsis* contain *AtbZIP9*, *AtbZIP10*, *AtbZIP25*, and *AtbZIP63. BZO2H1*/*AtbZIP10* is involved in pathogen-induced hypersensitive reactions, basal defense responses and reactive oxygen-induced cell death (Vincentz et al., 2003; Kaminaka et al., 2006). AtbZIP63 is important for the cross-talk between carbohydrate and ABA-regulated responses (Matiolli et al., 2011). AtbZIP25 is the orthologous protein of the maize OPAQUE2 bZIP factor, which regulates seed storage protein (SSP) genes (Lara et al., 2003). Five group C members from rice include *OsbZIP20*, *OsbZIP33*, *OsbZIP52*, *OsbZIP58*, and *OsbZIP88*. OsbZIP58 is involved in SSP synthesis, free lysine content, and starch biosynthesis (Yamamoto et al., 2006; Kawakatsu et al., 2009; Kawakatsu and Takaiwa, 2010; Wang et al., 2013). *OsbZIP20*, *OsbZIP33*, *OsbZIP52*, and *OsbZIP88* respond to abiotic stress. *OsbZIP52* can enhance sensitivity to cold and drought stress when overexpressed in rice (Nijhawan et al., 2008; Liu et al., 2012). In addition, an *OsbZIP52* orthologous gene, *ZmbZIP112* from maize, significantly increases its expression level under drought stress (Wei et al., 2012). Transgenic *Arabidopsis* plants overexpressing group C gene *GmbZIP62* are more tolerant to salt and freezing stresses compared with wild-type (WT) plants (Liao et al., 2008). However, in wheat, none of group the C-bZIP TFs or their abiotic functions have been reported.

To provide gene resources to improve wheat abiotic stress tolerance, we isolated a novel group C-bZIP TF, which we designated *TabZIP14-B*, from a wheat leaf full-length cDNA library. The expression of this gene was activated by exogenous ABA, polyethylene glycol (PEG), salt, and low-temperature treatments. TabZIP14-B could bind the ABRE *cis*-element *in vitro*. Furthermore, transformed *Arabidopsis* overexpressing *TabZIP14-B* exhibited improved freezing and salt tolerances and elevated plant sensitivity to ABA.

## Materials and Methods

### Cloning and Analysis of the *TabZIP14* Gene

Genomic sequences of *TabZIP14* were isolated from species of different ploidies, including *Triticum urartu* accession UR206 (A genome; Lebanon, Asia), *Aegilops speltoides* accession Y2006 (S genome; Syria, Asia), *Aegilops tauschii* accession Y2282 (D genome; Armenia, Asia), and Chinese Spring wheat (CS, ABD genome; Sichuan, China).

The complete nucleotide sequence of the *TabZIP14-B* gene was derived from a Chinese Spring wheat leaf full-length cDNA plasmid library. Gene-specific primers (forward 5′-CGATCAAACCCTCGAA-3′ and reverse 5′-CACAGCCAAAGAACAAA-3′) were designed to obtain the genomic sequences of *TabZIP14*. The alignment of the three *TabZIP14* homeologous genomic sequences with the corresponding cDNA sequences was used to determine the introns and exons of the genomic DNA sequences.

### Phylogenetic Tree Construction of Three *TabZIP14* Homeologous Proteins

C-bZIP sequences were acquired from wheat and other plant species through protein BLAST searches^1^. Amino acid sequence identity analysis was performed using the MegAlign program in DNASTAR. Phylogenetic analysis was carried out using MEGA 5.1 software.

### Isolation and Analysis of the *TabZIP14* Promoter

The promoter regions of three *TabZIP14* homeologous genes were acquired by comparing their genomic sequence with Chinese Spring genome reference^2^. These three promoter sequences were submitted to the PLACE database and analyzed to identify *cis*-acting elements^3^.

### SNP Discovery and Diversity Analysis of *TabZIP14-B* in Tetraploid and Hexaploid Wheat

A total of 213 wheat accessions, including 31 tetraploid species, 97 landraces, and 85 modern cultivars, were used to detect the SNPs and haplotypes of *TabZIP14-B* (Supplementary Table S1). The genomic sequence of *TabZIP14-B* was used to blast (*E*-value is 0.01) the 630,517 probe sequences from the wheat 660K SNP array, and then the matched probe sequences were selected to screen 213 wheat accessions for SNP discovery in the *TabZIP14-B* genomic sequence region. Standard statistical analyses of the genetic diversity of *TabZIP14-B*, including polymorphism information content (PIC) and gene diversity, were carried out using PowerMarker v3.25 (Liu and Muse, 2005).

### Real-Time PCR of Gene Expression

To detect the expression of three *TabZIP14* homeologous genes in response to PEG, NaCl and ABA treatments, germinated wheat seedlings were grown at 25°C in an artificial climate chamber with a 16 h light/8 h dark cycle. Ten-day-old seedlings of the Hanxuan 10 wheat cv. (drought resistant), the Chadianhong wheat cv. (salt-resistant) and CS were soaked in a 16.1% PEG-6000 solution, a 250 mM NaCl solution and a 200 μM ABA solution, respectively. Roots were sampled for expression analysis at 0, 1, 3, 6, 12, 24, and 48 h after treatment. For the low-temperature (4°C) treatment, the CS seedlings were transferred from normal growth conditions to a 4°C artificial climate chamber and leaves were harvested for expression analysis at 0, 1, 3, 6, 12, 24, and 48 h. Plant tissues (roots, stems, leaves, and young spikes) were obtained from CS wheat at the booting stage for tissue expression analysis of *TabZIP14-B*.

The expression levels of *AtRD29A*, *AtCOR47*, *AtRAB18*, *AtRD20*, and *AtGSTF6* were examined in transgenic plants. *Triticum aestivum glyceraldehyde-3-phosphate dehydrogenase* (*TaGAPDH*) (NCBI accession: AF251217.1) and *Arabidopsis thaliana Actin* (NCBI accession: NM_112764) were selected as internal references. RNA extraction and quantitative real-time PCR (RT-qPCR) were conducted in triplicate as previously described (Zhang et al., 2016). The 2^-ΔΔCT^ method was used to calculate the relative expression of the three *TabZIP14* homeologous genes. The specific gene primer sequences used in this experiment are provided in Supplementary Table S2.

### Subcellular Localization of the TabZIP14-B Protein

The open reading frame (ORF) of *TabZIP14-B* without the stop codon was obtained *via* PCR amplification using gene-specific primers. The purified PCR products were fused to the GFP-pEarleyGate vector containing the 35S promoter (35S::GFP-*TabZIP14-B*) using the gateway method. The empty 35S::GFP vector was used as the control. The 35S::GFP-TabZIP14-B fusion protein was introduced into *Agrobacterium tumefaciens* strain GV3101-pMP90, which was then transformed into epidermal cells of *N. benthamiana* (Sheludko et al., 2007). The transformed *N. benthamiana* leaves were cultivated for 2–6 days. Confocal laser scanning microscopy (Zeiss Lsm 700, Zeiss, Jena, Germany) was used to observe the transformed tobacco cells.

### Transcription Activation Assay in Yeast

The full-length *TabZIP14-B* cDNA was fused to the pDEST32 vector using the gateway method. The *pGAL4* and *pDEST32* vectors were used as positive and negative controls, respectively. The fused constructs were transformed into yeast strain AH109 with the His3 and Ade2 reporter genes. Transformed yeast cells were cultured on medium without histidine, leucine, and adenine to identify the transactivation abilities of the constructs.

### Assaying the DNA Binding Ability of the TabZIP14-B Protein Using the Yeast One-Hybrid System

The bait plasmid pRS315, which contains six copies of the ABRE/mABRE sequence and the *Leu* reporter gene, was provided by Professor Jun Zhao (Biotechnology Research Institute, Chinese Academy of Agricultural Sciences, Beijing, China). The full-length *TabZIP14-B* gene was introduced into the pDEST22 vector to generate the recombinant plasmid *pDEST22*- *TabZIP14-B*, which was used as the effector plasmid. The bait plasmid and effector plasmid were co-transformed into the yeast strain YM4271. The transformants were screened on SD/Leu^-^Trp^-^ at 30°C for approximately 2 days. The selected yeast cells were coated onto SD/Leu^-^Trp^-^His^-^ with 0.5 mM 3-AT to examine cell growth. The *pDEST22* vector and the bait plasmid used as the control were transformed into the YM4271 yeast strain, and the growth status of the transformants was checked.

### Generation and Phenotypic Analyses of Transgenic *Arabidopsis* Plants

The coding region of *TabZIP14-B* was ligated into the pEarleyGate 100 vector to obtain the 35S::*TabZIP14-B* recombinant plasmid, which was transferred into the GV3101-90RK *Agrobacterium tumefaciens* strain and then introduced into *A. thaliana* ecotype Col-0 *via* the floral dip method (Clough and Bent, 1998). The transgenic lines were sprayed with Basta solution and subsequently confirmed *via* RT-qPCR to screen homozygous lines.

For the freezing tolerance assay, WT and transgenic *Arabidopsis* seedlings were grown in potted soil for approximately 2 weeks, after which the plants were transferred to -10°C for 3 h. The plants were subsequently placed at 5°C for 2 h and then transferred back to normal conditions.

For the NaCl tolerance assay, WT and transgenic *Arabidopsis* seeds were transferred from Murashige and Skoog (MS) plates to the potted soil and well watered. Water was halted for 3 weeks, and the seedlings were watered with NaCl solution (250 mM) from the bottom of the plates. When the volume of the NaCl solution no longer reduced, the NaCl solution was removed, and the plants were grown under normal conditions.

To study the response of the transgenic plants to ABA, WT and transgenic *Arabidopsis* seeds were planted in MS medium for 5 days, transferred to new MS medium with 0 or 24 μM ABA and cultured vertically for 7 days. After that period, the root lengths of the plants were assessed. All experiments were repeated independently three times.

### Measurements of the Proline Content, Soluble Sugar Content, and Relative Electrolyte Leakage

Free proline concentrations were determined based on a previously described method (Zhang et al., 2015). Electrolyte leakage and the soluble sugar content were measured according to a method described previously (Lehner et al., 2006; Cao et al., 2007).

All measurements were conducted in triplicate, and Student’s *t*-test was performed for statistical analysis.

## Results

### *TabZIP14* Isolation and Analysis

The complete *TabZIP14-B* nucleotide sequence was obtained by sequencing a cDNA library. Sequence analysis showed that *TabZIP14-B* contained a 1576 bp gene fragment encoding a polypeptide of 400 amino acids including the conserved bZIP domain (279–393 aa), with a predicted molecular mass of 41.9 kDa and a *pI* of 5.06. The *TabZIP14-B* gene was submitted to GenBank under accession number KY113325.

**FIGURE 1:**
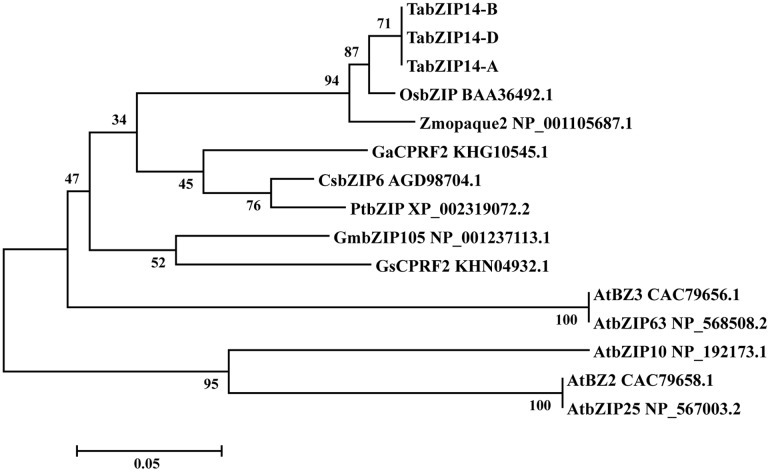
**Phylogenetic relationships of selected group C-bZIP proteins and the three TabZIP14 homeologous proteins**.

To assay the molecular structures of the *TabZIP14* homeologous genes in wheat, we isolated the genomic sequences from diploid wheat genomes A (UR206), S (Y2006), and D (Y2282) and three homeologous sequences from the hexaploid CS wheat. After comparison with the diploid *TabZIP14* genomic sequences, the three hexaploid sequences from subgenomes A, B and D of hexaploid wheat were determined and denoted as *TabZIP14-A*, *TabZIP14-B*, and *TabZIP14-D*, respectively. To determine the gene structure of the *TabZIP14*, the cDNA sequences of each *TabZIP14* gene were aligned with the gDNAs. The results indicated that the three homeologous genes all contained six exons and five introns; the intron length varied, specifically for intron 1, which varied from 1159 to 1709 bp; and the exon size was relatively conserved, with exons 2, 3, and 5 having the same lengths (Supplementary Table S3). *TabZIP14-A*, *TabZIP14-B*, and *TabZIP14-D* showed 94.3–96.2% similarity at the amino acid level. They grouped together in one group (**Figure 1**). Phylogenetic analysis with other species indicated that TabZIP14 homeologous proteins formed a phylogenetic tree with the members group C of the bZIP family. For instance, the amino acid sequence alignment showed that TabZIP14-B shared 72% identity with Zmopaque2 (NP_001105687.1), 41.4% identity with AtbZIP63 (NP_568508.2), 31.5% identity with AtbZIP25 (NP_567003.2), and 28.4% identity with AtbZIP10 (NP_192173.1) (**Figure 1**).

The 1800-bp promoter sequences of three *TabZIP14* homeologous genes were obtained and analyzed for *cis*-acting regulatory elements individually. Seven *cis*-elements, including TATA-box, CAAT-box, G-box, GCN4-motif, LTR, MBS, and P-box, were identified in all three promoter regions. In addition to the seven *cis*-elements, O2-site involved in zein metabolism regulation was detected in the promoter region of *TabZIP14-A*, ERE involved in ethylene-responsive element was found in the promoter region of *TabZIP14-B*, and ABRE *cis*-element involved in ABA responsiveness was present in the promoter region of *TabZIP14-D* (Supplementary Table S4).

Genomic sequence variations of *TabZIP14-B* among 213 wheat accessions were also analyzed in the present study. Three SNPs were found in *TabZIP14-B*, including two in the first intron and one in the third exon, and three haplotypes, denoted Hap1, Hap2, and Hap3, were formed based on these three SNPs (**Supplementary Figure S1A**). Haplotype frequency in tetraploids, landraces, and modern cultivars showed that Hap1 increased but Hap2 decreased from tetraploids to landraces, while the pattern was opposite from landraces to modern cultivars (**Supplementary Figure S1B**). Moreover, gene diversity of *TabZIP14-B* was significantly reduced in landraces and modern cultivars compared with that in tetraploid species (**Supplementary Figure S1C**).

### Expression Patterns of *TabZIP14* in Different Organs of Wheat and Responses to Abiotic Stresses

The tissue expression patterns of *TabZIP14-B* were analyzed *via* RT-qPCR. *TabZIP14-B* was expressed in all of the analyzed tissues, including the roots, stems, leaves, and young spikes. The relative expression levels were highest in the leaves (**Supplementary Figure S2**).

**FIGURE 2:**
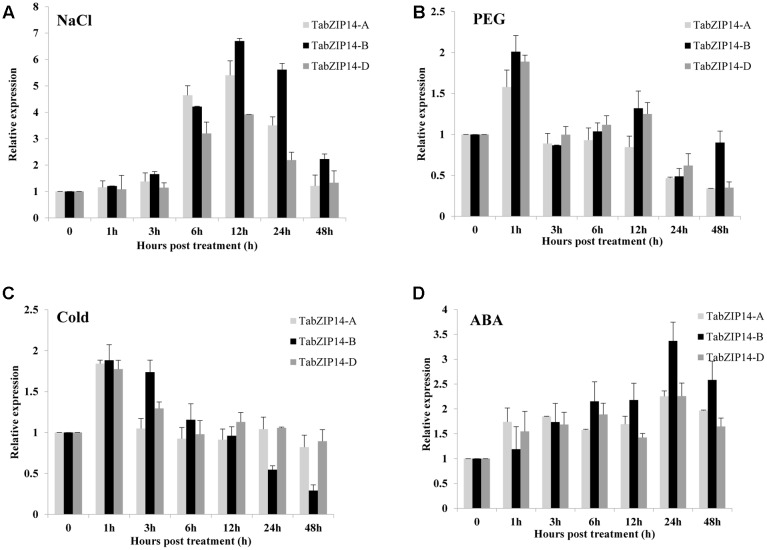
**Expression patterns of three *TabZIP14* homeologous genes under different abiotic stresses.** *TaGAPDH* was used as the control. The 2^-ΔΔCT^ method was used to calculate the relative expression of the three *TabZIP14* homeologous genes. **(A–D)** Expression patterns of *TabZIP14* following salinity, PEG, low temperature (4°C) and ABA treatments. The bars indicate the standard errors.

The expression levels of three *TabZIP14* homeologous genes in wheat seedlings exposed to PEG, NaCl, ABA, and low temperature were also tested using the RT-qPCR method. The results showed that *TabZIP14-A*, *TabZIP14-B*, and *TabZIP14-D* had similar expression patterns. The expression levels of three *TabZIP14* homeologous genes began to increase for 3 h and reached their highest levels 12 h after exposure to NaCl treatment, and the expression level of *TabZIP14-B* was higher than that of *TabZIP14-A* and *TabZIP14-D* 12 h post-treatment (**Figure 2A**). Under PEG and low temperature stresses, the expression of three *TabZIP14* homeologous genes was induced and peaked 1 h post-treatment (**Figures 2B,C**). In addition, the expression of the three *TabZIP14* homeologous genes was also induced under ABA conditions and peaked 24 h post-treatment (**Figure 2D**), and the expression level of *TabZIP14-B* was higher than that of *TabZIP14-A* and *TabZIP14-D* 24 h post-treatment.

Given that the expression patterns of the three *TabZIP14* homeologous genes in wheat were similar in response to abiotic stress and that *TabZIP14-B* was obtained from a wheat full-length cDNA library, *TabZIP14-B* was chosen for further study.

### Subcellular Localization of the TabZIP14-B Protein

**FIGURE 3:**
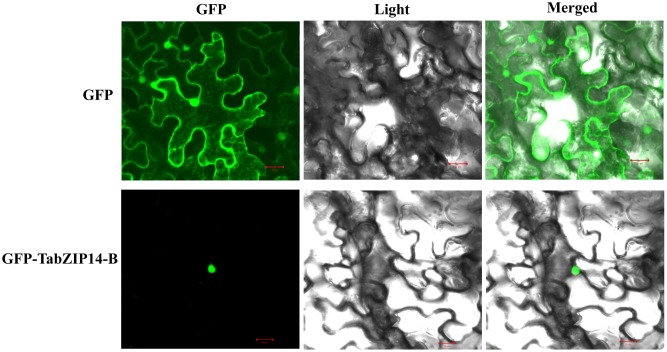
**Subcellular localization of the TabZIP14-B protein.** A vector harboring *GFP* or *GFP-TabZIP14-B* was transferred into tobacco leaves. Bars = 20 μM.

We checked the subcellular localization of the TabZIP14-B protein and found that the cells transformed with 35S::GFP alone exhibited green fluorescence signals in both the cytoplasm and the nucleus. However, the expression of 35S::GFP-TabZIP14-B was only observed in the nucleus (**Figure 3**). These results demonstrated that the TabZIP14-B protein was localized to the nuclei.

### Transcriptional Activation of TabZIP14-B

**FIGURE 4:**
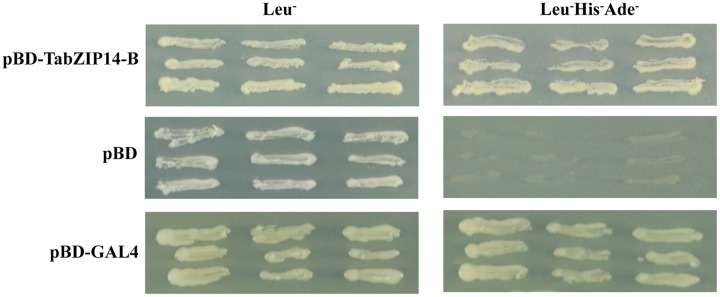
**Transcriptional activation activity of the TabZIP14-B protein.** The *pDEST32* vector alone and *GAL4* were used as the negative and positive controls, respectively.

The transactivation abilities of TabZIP14-B were analyzed using a yeast assay system. All transformants grew well on SD plates with SD/Leu^-^ medium. The yeast that expressed full-length *TabZIP14-B* and *pGAL4* grew on SD plates with SD/Leu^-^His^-^Ade^-^ medium, but the yeast harboring the *pDEST32* vector alone failed to grow on SD/Leu^-^His^-^Ade^-^ medium (**Figure 4**). These results indicated that the TabZIP14-B protein could activate the transcription of the reporter genes *Ade* and *His* in the yeast system, which suggests that the TabZIP14-B protein exhibits transcriptional activation capability.

### The TabZIP14-B Protein Specifically Binds to the ABRE *Cis*-Element in Yeast Cells

**FIGURE 5:**
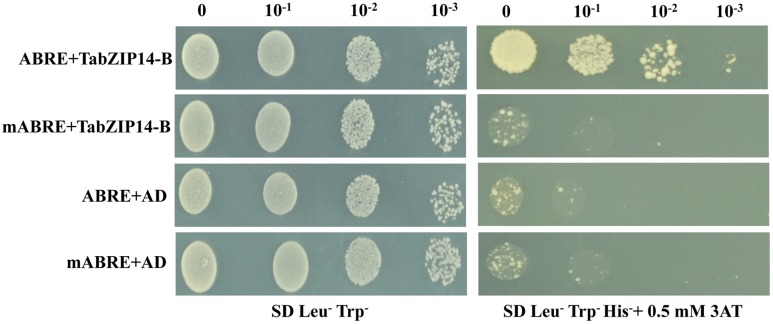
**Analysis of the ABRE binding activity of the TabZIP14-B protein.** mABRE+TabZIP14-B, mABRE+AD, and ABRE+AD were used as negative controls. Transformed yeast cells were coated onto SD/Leu^-^Trp^-^ and on SD/Leu^-^Trp^-^His^-^ medium with 0.5 mM 3-AT in a 10x dilution series.

Plant bZIP proteins bind to DNA sequences with an ACGT core *cis*-element. We examined whether the TabZIP14-B protein could bind to the ABRE *cis*-element using the yeast one-hybrid assay system. **Figure 5** demonstrates that all of the transformants grew on medium containing SD/Leu^-^Trp^-^. However, only the transformants harboring the effector plasmid pAD-TabZIP14-B and the bait plasmid with the ABRE *cis*-element were able to grow on SD/Leu^-^Trp^-^His^-^ containing 3-AT medium, which suggests that the TabZIP14-B protein had an affinity for the ABRE *cis*-element.

### Overexpression of *TabZIP14-B* in *Arabidopsis* Affects ABA Sensitivity

We transformed *Arabidopsis* with *TabZIP14-B* and generated more than 10 transgenic lines. The expression levels of lines 1, 2, and 3 (denoted L1, L2, and L3, respectively) are shown in **Supplementary Figure S3**. These three lines were used to study gene function.

**FIGURE 6:**
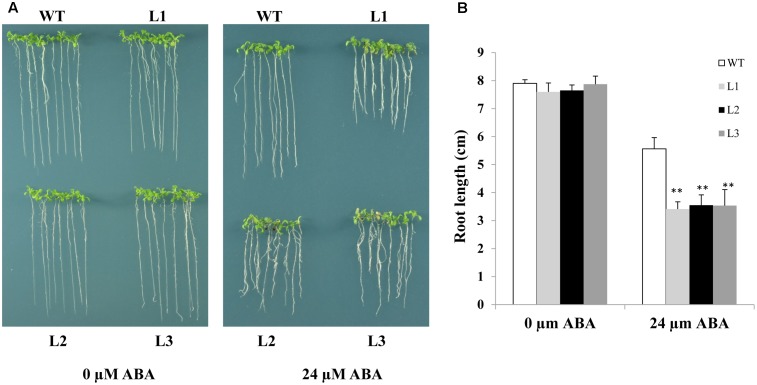
**Overexpression of *TabZIP14-B* in *Arabidopsis* affects ABA sensitivity.** **(A)** Comparisons of root length on MS or MS with different concentrations of ABA between WT and transgenic plants. **(B)** Statistical analyses of root length. Three independent experiments were executed. The bars indicate the SD. ^∗^ and ^∗∗^ represent significant differences compared with the WT lines at the 0.01 < *P* < 0.05 and *P* < 0.01 levels.

As *TabZIP14-B* expression is induced by ABA, we assessed whether overexpression of *TabZIP14-B* in *Arabidopsis* affects ABA sensitivity. ABA sensitivity was scored by measuring root length in transgenic and WT plants in the presence of 24 μM ABA for 7 days. The root growth of the transgenic plants was hindered more severely compared with that of the WT plants. This result indicated that the overexpression of *TabZIP14-B* resulted in severe ABA sensitivity and that TabZIP14-B participated in the ABA signaling pathway (**Figure 6**).

### Overexpression of *TabZIP14-B* Increases Freezing and Salt Tolerance

**FIGURE 7:**
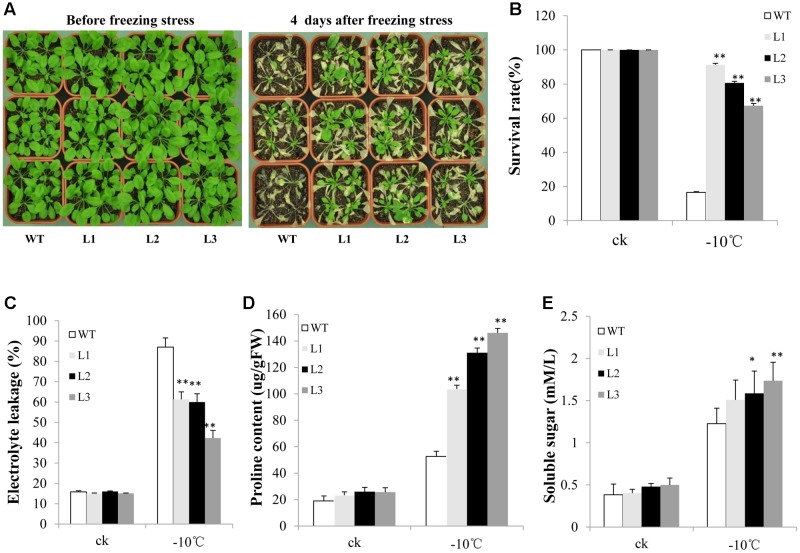
**Overexpression of *TabZIP14-B* in *Arabidopsis* confers freezing tolerance.** **(A)** Freezing tolerance phenotypes of WT and transgenic plants measured before and after freezing treatments. **(B)** Survival rates of the seedlings in **(A)**. **(C)** Relative electrolyte leakage. **(D)** Proline content. **(E)** Soluble sugar content. Three independent experiments were performed. The bars indicate the SD. ^∗^ and ^∗∗^ represent significant differences compared with the WT lines at the 0.01 < *P* < 0.05 and *P* < 0.01 levels.

Many studies have indicated that group C-bZIP TFs are involved in freezing and salt tolerance. Therefore, we evaluated the ability of *TabZIP14-B* transgenic plants to resist abiotic stress. After treatment at -10°C for 3 h, nearly 90% of the WT seedlings were dead. However, the survival rates of the transgenic lines were approximately 60–90% (**Figures 7A,B**). Furthermore, electrolyte leakage in the transgenic plants was lower than in the WT plants when the plants were subjected to freezing stress (**Figure 7C**). The transgenic plants engendered obviously high levels of proline content compared with those of WT plants under freezing conditions (**Figure 7D**). However, the soluble sugar content changed little before and after freezing treatment (**Figure 7E**).

**FIGURE 8:**
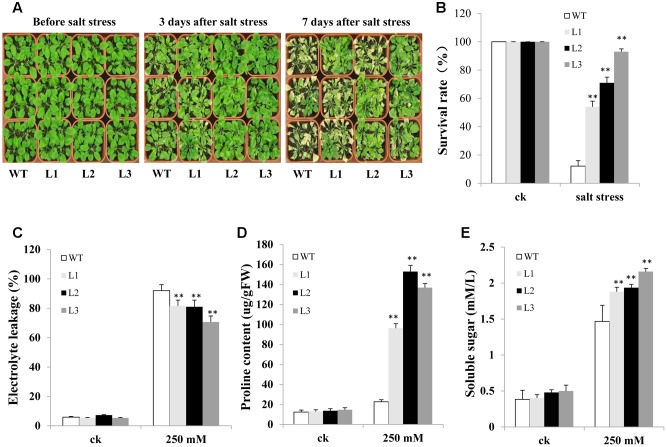
**Overexpression of *TabZIP14-B* in *Arabidopsis* strengthens salt tolerance.** **(A)** Salt tolerance phenotypes of WT and transgenic plants measured before and after salt treatments. **(B)** Survival rate of the seedlings in **(A)**. **(C)** Relative electrolyte leakage. **(D)** Proline content. **(E)** Soluble sugar content. Three independent experiments were carried out. The bars indicate the SD. ^∗^ and ^∗∗^ represent significant differences compared with the WT lines at the 0.01 < *P* < 0.05 and *P* < 0.01 levels.

We also evaluated salt resistance between *TabZIP14-B* transgenic *Arabidopsis* and WT plants in the soil. There were no significant differences between WT and transgenic plants under normal conditions. However, when the *Arabidopsis* seedlings and WT plants were treated with 250 mM NaCl solution, 3 days later, most of the leaves of WT plants began to turn yellow and shrink. However, no evident shrinking was observed for most transgenic *Arabidopsis* seedlings. The transgenic lines looked greener than did the WT plants. Seven days later, most of the WT plants were dead. The survival rates of transgenic seedlings were 54–93%, significantly higher than in the WT plants (**Figures 8A,B**). In addition, after salt stress, *TabZIP14-B*-overexpressing lines exhibited lower electrolyte leakage than did WT plants (**Figure 8C**). Moreover, the proline content and soluble sugar levels were significantly higher in *TabZIP14-B*-overexpressing plants than in the WT plants (**Figures 8D,E**).

### TabZIP14-B Regulates the Expression of Stress-Responsive Genes

As the transgenic plants showed enhanced salt and freezing tolerance, we investigated whether abiotic stress-responsive genes were altered in the *TabZIP14-B*-transgenic plants under salt stress. As shown in **Supplementary Figure S4**, the expression levels of *AtRD29A*, *AtCOR47*, *AtRD20*, *AtGSTF6*, and *AtRAB18* were enhanced in *TabZIP14-B* transgenic plants compared with WT plants. The promoter regions of these up-regulated genes were obtained using the *Arabidopsis* genome database. Our results showed that these genes also contained the bZIP ACGT-element within their promoter regions, suggesting that *TabZIP14-B* may confer stress tolerance by regulating various stress-responsive genes.

## Discussion

TabZIP14-B isolated in this study belongs to the C-bZIP family. The promoter region of *TabZIP14-B* carried the abiotic stress-responsive elements LTR and MBS, the biotic stress-related elements TCA and ERE, and light-responsive elements. These regulatory elements suggest that TabZIP14-B is involved in plant development and stress tolerance; *TabZIP14-B* was up-regulated when wheat seedlings were stressed by salt, low-temperature, PEG and ABA treatments, and overexpression of *TabZIP14-B* could increase plant tolerance to salt and freezing but could not yield drought tolerance. This result is consistent with previous reports on other C-bZIP TFs, such as GmbZIP62 (Liao et al., 2008), possibly because *TabZIP14-B* is heterologously expressed in *Arabidopsis* plants. The drought tolerance of *Arabidopsis* overexpressing *TabZIP14-B* might be achieved through TabZIP14-B interactions with other protein or TFs binding to the *cis*-elements in the promoters of drought-responsive genes, which might not be present in *Arabidopsis*. However, most of the group A-bZIP genes have been found to participate in drought tolerance and ABA signaling (Zhang et al., 2015; Li et al., 2016; Wang et al., 2016). This difference indicates the functional specificity of each bZIP gene in regulating plant stress resistance, and each group of bZIP genes has a particular function. The survival rates of the transgenic lines were higher compared with those of WT under salt and freezing stress (**Figures 7**, **8**), but there were differences among L1, L2, and L3 in the survival rates. This difference may be due to the different insertions of the *TabZIP14-B* in *Arabidopsis* genome in the three transgenic lines.

To a large extent, the enhancement of abiotic stress resistance can be attributed to changes in physiological parameters, including soluble sugar and proline contents and electrolyte leakage. Proline is an osmolyte that plays a significant role in stress tolerance (Hayat et al., 2012). In this study, *TabZIP14-B*-transgenic plants showed significantly higher levels of proline compared with those of WT plants under freezing and salt treatments. These findings are consistent with those of a previous report on the TF GmbZIP62 (Liao et al., 2008). Soluble sugars are the main osmotic regulators in many plants. As the concentration of soluble sugar increases under abiotic stress, transgenic plants also showed more adaptation to stress compared with WT plants (Lecourieux et al., 2014). In this work, the soluble sugar content of WT plants was lower than that in *TabZIP14-B*-transgenic plants subjected to freezing and salt treatments.

Many bZIP TFs are capable of binding to ABRE *cis*-elements (Uno et al., 2000), and group C-bZIP TFs can exhibit different responses to ABA. For example, *OsbZIP52*-overexpression plants are not responsive to ABA treatment (Liu et al., 2012). Overexpression of the *GmbZIP62* gene results in reduced sensitivity to ABA (Liao et al., 2008). In addition, in the present study, plants overexpressing *TabZIP14-B* appeared to be more sensitive to ABA compared with WT plants. Moreover, the *TabZIP14-B* plants exhibited induction at the transcriptional level following ABA treatment. These results showed that TabZIP14-B functions as positive regulator of ABA signaling. Furthermore, we determined that plants overexpressing *TabZIP14-B* presented up-regulated expression levels of stress-responsive genes, including *AtRD29A*, *AtCOR47*, *AtRD20*, *AtGSTF6*, and *AtRAB18.* These genes participate in multiple stress responses. ABRE and ABRE-like *cis*-elements have been identified in the promoter sequence of the above five genes (Uno et al., 2000; Liu et al., 2008; Aubert et al., 2010; Hsieh et al., 2010; Gao et al., 2011; Lan et al., 2013). *AtRD20* belongs to the caleosin family, and its expression is enhanced by ABA, salt, dehydration, and osmotic stresses (Aubert et al., 2010). *AtCOR47* and *AtRAB18* belong to the dehydrin protein family. *AtRAB18* and *AtCOR47* are well-known stress- and ABA-responsive genes (Puhakainen et al., 2004). *AtRD29A* gene expression is induced by dehydration, salt, cold, and ABA treatment (Narusaka et al., 2003). GSTF6 encodes glutathione transferase and is involved in oxidative and salt stress (Lan et al., 2013). The ABRE *cis*-elements may mediate the TabZIP14-regulated expression of the *AtRD29A*, *AtCOR47*, *AtRD20*, *AtGSTF6*, and *AtRAB18* genes. These results show that TabZIP14-B is involved in the abiotic stress responses *via* an ABA-dependent pathway.

Here, we report the novel wheat group C-bZIP gene *TabZIP14-B*, which is induced by salt, low temperature, PEG, and ABA. In yeast, TabZIP14-B acted as a transcriptional activator and could bind to the ABRE *cis*-element. Ectopic overexpression of *TabZIP14-B* in *Arabidopsis* conferred freezing and salt stress tolerances and improved sensitivity to ABA through the regulation of the expression of stress-associated genes and changes in physiological indexes. The novel characteristic of TabZIP14 showed the different roles of each bZIP group of TFs in drought response as well as their difference between *Arabidopsis* and wheat. Functional research on TabZIP14 offers an opportunity to clarify the differences in the mechanisms underlying tolerance to drought stresses.

## Author Contributions

LNZ, LCZ, CX, LFG, CYH, and GYZ carried out most of the experiments. LNZ, LCZ, JZJ, and XYK designed the experiments and wrote this paper. All authors read and approved the final manuscript.

## Conflict of Interest Statement

The authors declare that the research was conducted in the absence of any commercial or financial relationships that could be construed as a potential conflict of interest.

## Footnotes

1 http://blast.ncbi.nlm.nih.gov/Blast.cgi

2 https://urgi.versailles.inra.fr/blast/blast.php

3 http://bioinformatics.psb.ugent.be/webtools/plantcare/html/

## Supplementary Material

**FIGURE S1 | Genetic diversity analysis of *TabZIP14-B*.** **(A)** Three SNPs of the *TabZIP14-B*. Solid blocks represent exons; lines between exons indicate introns. The positions of three SNPs are shown on the genomic sequence, and three Haplotypes are listed below; **(B)** the frequencies of three Haplotype in tetraploid, landraces, and modern cultivars are shown in percentages; **(C)** genetic diversity of *TabZIP14-B* was evaluated with polymorphism information content (PIC) and gene diversity.

**FIGURE S2 | Expression of *TabZIP14-B* in different tissues as determined by RT-qPCR (R, root; S, stem; L, leaf; YS, young spike).** The expression of *TabZIP14-B* in stem tissues was regarded as the standard due to its low level.

**FIGURE S3 | RT-qPCR detection of the expression levels of *TabZIP14-B* in transgenic *Arabidopsis* lines.** The expression of *TabZIP14-B* in L5 was regarded as the standard due to its lower level.

**FIGURE S4 |*TabZIP14-B* modulates the expression of stress-responsive genes.** RT-qPCR was used to check the relative transcript levels of the stress-responsive genes.
