# Supplementary figures and images for: A Novel Wheat C-bZIP Gene, TabZIP14-B, Participates in Salt and Freezing Tolerance in Transgenic Plants
Source: Front Plant Sci. 2017 May 9;8:710. doi: 10.3389/fpls.2017.00710 (PMC5422549; doi:10.3389/fpls.2017.00710)

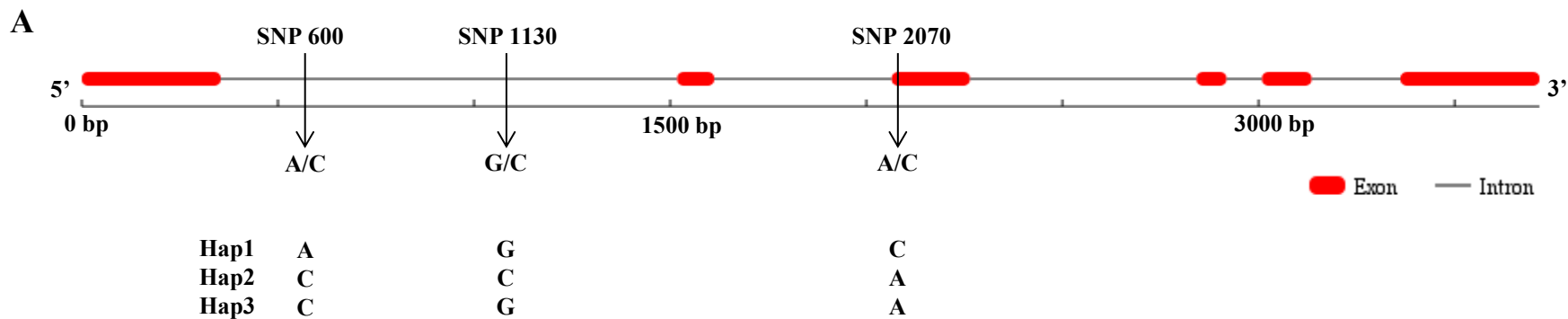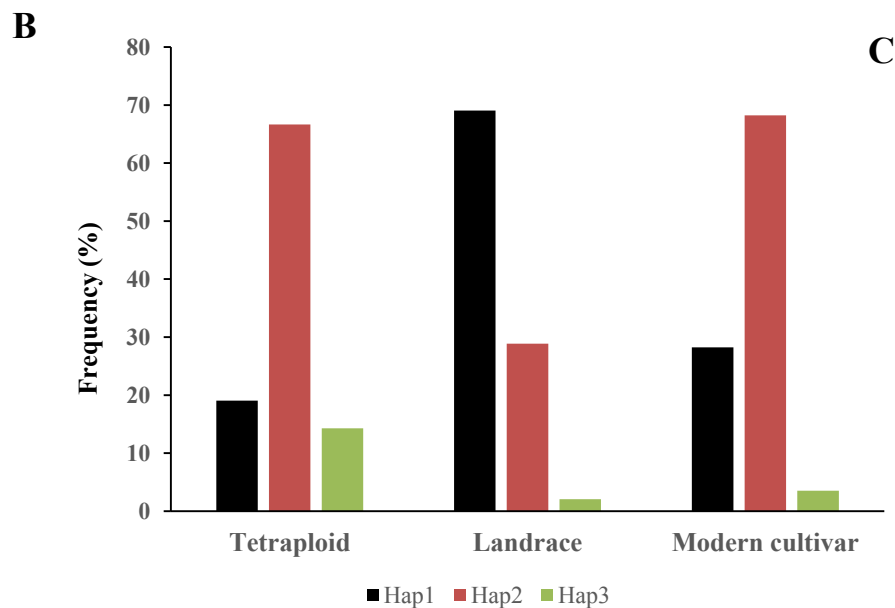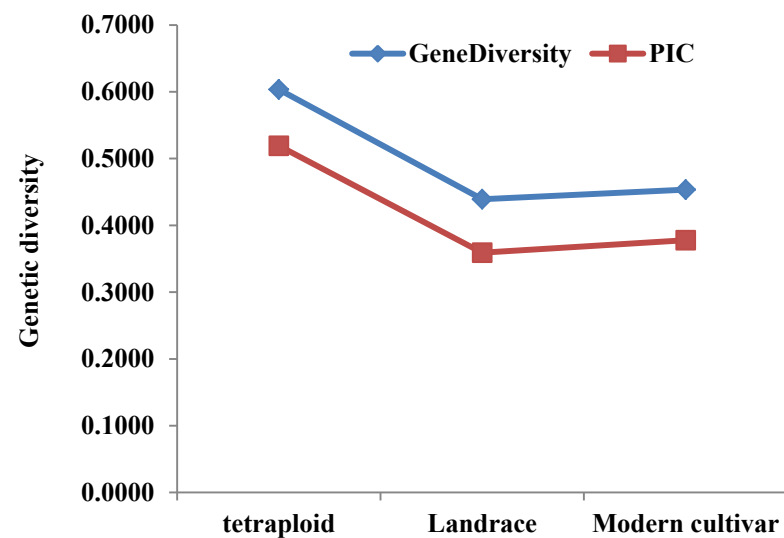

Supplement: Supplementary file 1 [file Image_1.PDF]

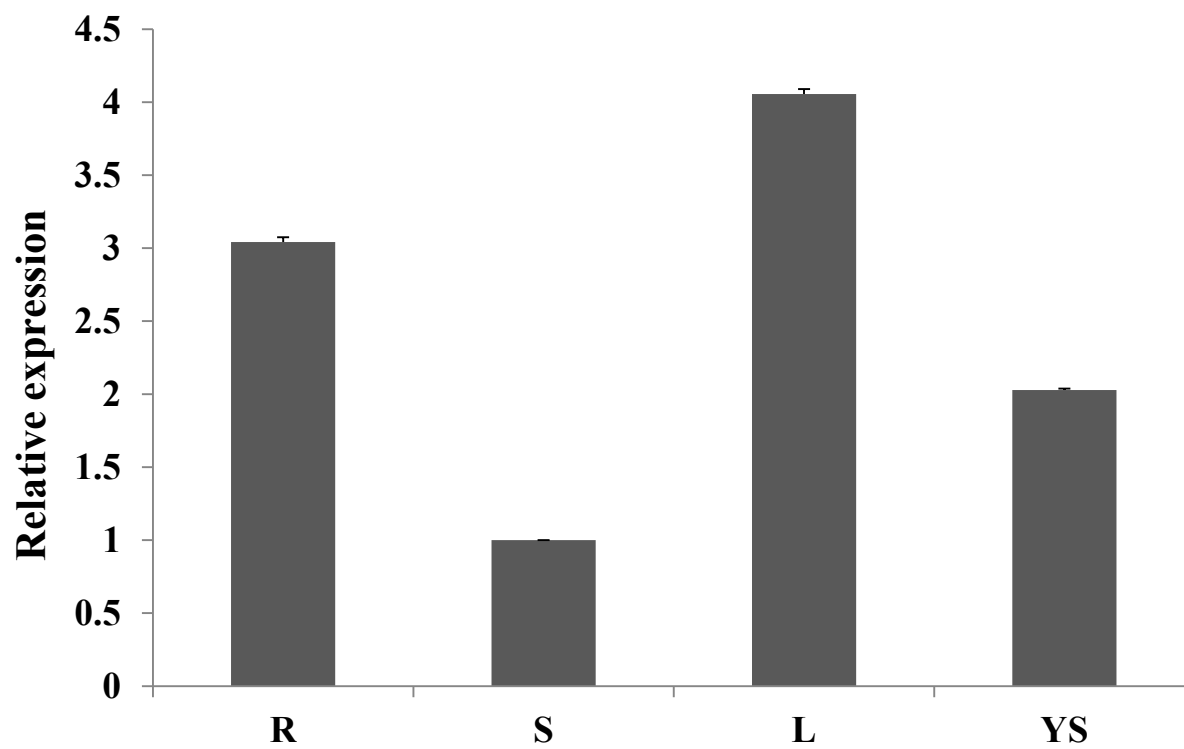

Supplement: Supplementary file 2 [file Image_2.PDF]

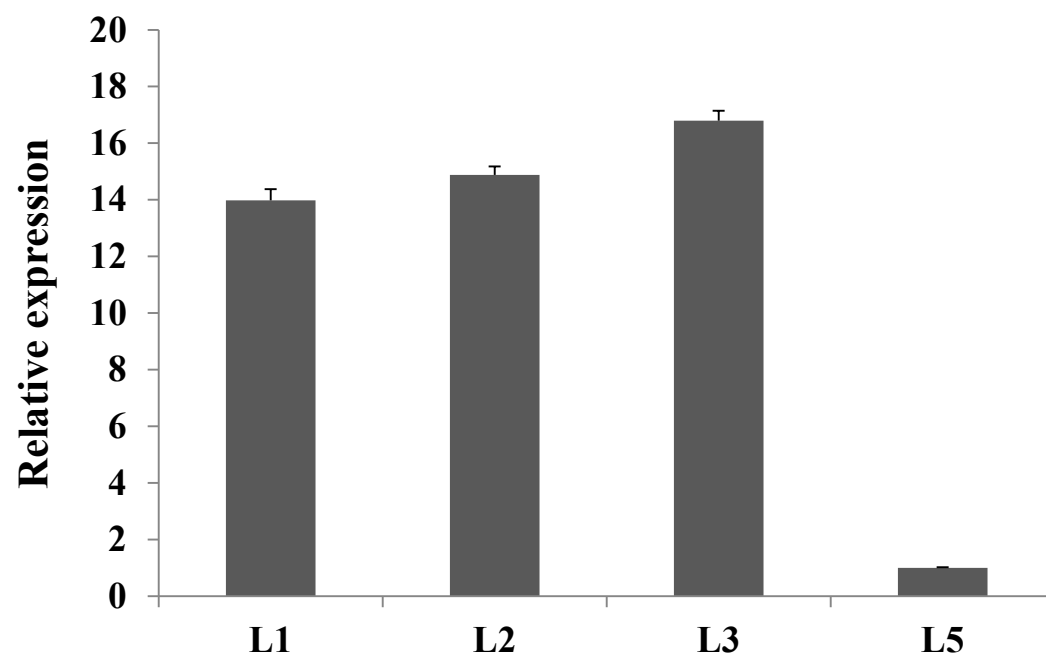

Supplement: Supplementary file 3 [file Image_3.PDF]

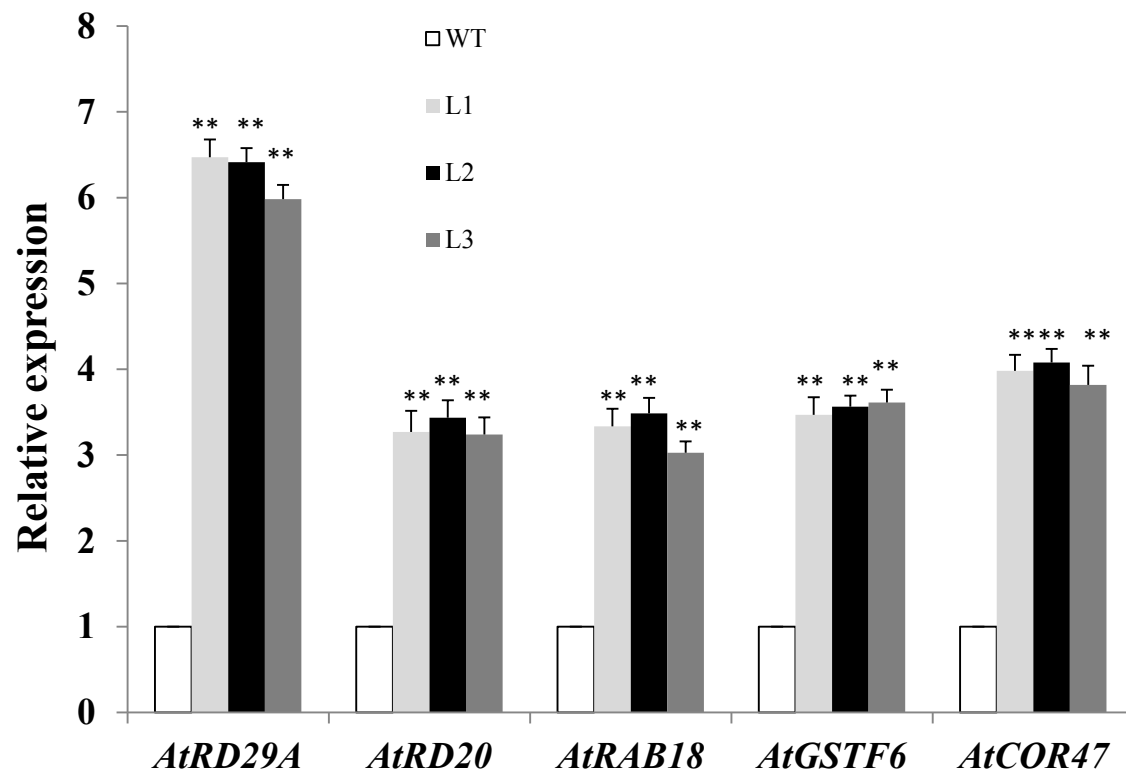

Supplement: Supplementary file 4 [file Image_4.PDF]
