# Supplementary material for: A Novel Wheat C-bZIP Gene, TabZIP14-B, Participates in Salt and Freezing Tolerance in Transgenic Plants
Source: Front Plant Sci. 2017 May 9;8:710. doi: 10.3389/fpls.2017.00710 (PMC5422549; doi:10.3389/fpls.2017.00710)
Supplement: Supplementary file 6 [file Table_2.DOCX]

**Supplementary Material**

Table 2. Primers used to detect gene expression via RT-qPCR

| Gene name | Accession | Forward primers(5'-3') | Reverse primers(5'-3') |
| --- | --- | --- | --- |
| *TaGAPDH* | AF251217 | TTAGACTTGCGAAGCCAGCA | AAATGCCCTTGAGGTTTCCC |
| *AtActin* | NM_112764 | CTCATGCCATCCTCCGTCTT | ACTTGCCCATCGGGTAATTC |
| *AtRD29A* | NM_124610 | GAGCAACGAGGGGAAGATAAAAG | TCAGTCGCACCACCACCGAACCA |
| *AtRAB18* | NM_126038 | GAGGAGGAAGAAGGGAATAACA | AAAAGCACAATACAACGACCGA |
| *AtRD20* | NM_128898 | ATTCGAGCACCTATGACACC | AAACTTCCATCAAAGCAACC |
| *AtCOR47* | NM_101894 | TCCCAGGACACCACGACAAGAC | CCTCTTCAGTGGTCTTGGCATG |
| *AtGSTF6*  *TabZIP14-A*  *TabZIP14-B*  *TabZIP14-D* | NM_100174 | ACTCTTCGACGAGCGTCCACAT  TGGCAGAGGACTCAGTGAAGCG  CAAAGACTGCGGAGGAGGAAGC  GAAAGGCAGCTCACCTGAATGA | GGTCATCGCCACTTTTATTACA  ATCTCAGGCATGTACCCGTTGTT  TGTCAACGGCAGCACCATTGTAT  CCTATTGTCAACAGCAGCACCA |
