# Supplementary material for: A Novel Wheat C-bZIP Gene, TabZIP14-B, Participates in Salt and Freezing Tolerance in Transgenic Plants
Source: Front Plant Sci. 2017 May 9;8:710. doi: 10.3389/fpls.2017.00710 (PMC5422549; doi:10.3389/fpls.2017.00710)
Supplement: Supplementary file 7 [file Table_3.DOCX]

**Supplementary Material**

Table 3. The sequence lengths of the introns and exons of three *TabZIP14* homeologous genomic sequences

| No | *TabZIP14-A* | | *TabZIP14-B* | | *TabZIP14-D* | |
| --- | --- | --- | --- | --- | --- | --- |
|  | Length of exon (bp) | Length of intron (bp) | Length of exon (bp) | Length of intron (bp) | Length of exon (bp) | Length of intron (bp) |
|  |  |  |  |  |  |  |
| 1 | 330 | 1709 | 354 | 1164 | 375 | 1159 |
| 2 | 94 | 454 | 94 | 453 | 94 | 455 |
| 3 | 199 | 593 | 199 | 578 | 199 | 574 |
| 4 | 76 | 89 | 76 | 91 | 94 | 89 |
| 5 | 126 | 200 | 126 | 227 | 126 | 199 |
| 6 | 354 |  | 354 |  | 351 |  |
