# Supplementary material for: A Novel Wheat C-bZIP Gene, TabZIP14-B, Participates in Salt and Freezing Tolerance in Transgenic Plants
Source: Front Plant Sci. 2017 May 9;8:710. doi: 10.3389/fpls.2017.00710 (PMC5422549; doi:10.3389/fpls.2017.00710)
Supplement: Supplementary file 8 [file Table_4.DOCX]

**Supplementary Material**

Table 4. The *cis*-acting elements in the promoter region of *TabZIP14-A*

| Name | Number | Sequence function |
| --- | --- | --- |
| TATA-box | 15 | core promoter element around -30 of transcription start |
| CAAT-box | 18 | common *cis*-acting element in promoter and enhancer regions |
| G-box | 6 | *cis*-acting regulatory element involved in light responsiveness |
| GCN4-motif | 2 | *cis*-regulatory element involved in endosperm expression |
| LTR | 2 | *cis*-acting element involved in low-temperature responsiveness |
| MBS | 2 | MYB binding site involved in drought-inducibility |
| O2-site | 1 | *cis*-acting regulatory element involved in zein metabolism regulation |
| P-box | 1 | the gibberellin-responsive element |
| TCA-element | 1 | *cis*-acting element involved in salicylic acid responsiveness |
| GATA-motif | 2 | light responsive element |

The *cis*-elements were identified in the upstream of promoters (1800bp)

The *cis*-acting elements in the promoter region of *TabZIP14-B*

| Name | Number | Sequence function |
| --- | --- | --- |
| TATA-box | 11 | core promoter element around -30 of transcription start |
| CAAT-box | 14 | common *cis*-acting element in promoter and enhancer regions |
| CGTCA-motif | 2 | *cis*-acting element involved in the MeJA-responsiveness |
| G-box | 1 | *cis*-acting regulatory element involved in light responsiveness |
| GCN4-motif | 3 | *cis*-regulatory element involved in endosperm expression |
| LTR | 2 | *cis*-acting element involved in low-temperature responsiveness |
| MBS | 2 | MYB binding site involved in drought-inducibility |
| P-box | 1 | the gibberellin-responsive element |
| TCA-element | 2 | *cis*-acting element involved in salicylic acid responsiveness |
| ERE | 1 | *cis*-acting involved in ethylene-responsive element |
| GATA-motif | 3 | light responsive element |

The *cis*-elements were identified in the upstream of promoters (1800bp)

The *cis*-acting elements in the promoter region of *TabZIP14-D*

| Name | Number | Sequence function |
| --- | --- | --- |
| TATA-box | 11 | core promoter element around -30 of transcription start |
| CAAT-box | 8 | common *cis*-acting element in promoter and enhancer regions |
| CCAAT-box | 1 | MYBHv1 binding site |
| TGACG-motif | 2 | *cis*-acting element involved in the MeJA-responsiveness |
| G-box | 4 | *cis*-acting regulatory element involved in light responsiveness |
| GCN4-motif | 2 | *cis*-regulatory element involved in endosperm expression |
| LTR | 2 | *cis*-acting element involved in low-temperature responsiveness |
| MBS | 1 | MYB binding site involved in drought-inducibility |
| P-box | 1 | the gibberellin-responsive element |
| GATA-motif | 1 | light responsive element |
| ABRE | 1 | cis-acting element involved in the abscisic acid responsiveness |

The *cis*-elements were identified in the upstream of promoters (1800bp)
